# Constraints on the onset duration of the Paleocene–Eocene Thermal Maximum

**DOI:** 10.1098/rsta.2017.0082

**Published:** 2018-09-03

**Authors:** Sandra Kirtland Turner

**Affiliations:** Department of Earth Sciences, University of California, Riverside, CA 92521, USA

**Keywords:** Paleocene–Eocene Thermal Maximum, earth system modelling, carbon emissions

## Abstract

The Paleocene–Eocene Thermal Maximum (PETM, approx. 56 Ma) provides a test case for investigating how the Earth system responds to rapid greenhouse gas-driven warming. However, current rates of carbon emissions are approximately 10 Pg C yr^−1^, whereas those proposed for the PETM span orders of magnitude—from ≪1 Pg C yr^−1^ to greater than the anthropogenic rate. Emissions rate estimates for the PETM are hampered by uncertainty over the total mass of PETM carbon released as well as the PETM onset duration. Here, I review constraints on the onset duration of the carbon isotope excursion (CIE) that is characteristic of the event with a focus on carbon cycle model-based attempts that forgo the need for a traditional sedimentary age model. I also review and compare existing PETM carbon input scenarios employing the Earth system model cGENIE and suggest another possibility—that abrupt input of an isotopically depleted carbon source combined with elevated volcanic outgassing over a longer interval can together account for key features of the PETM CIE.

This article is part of a discussion meeting issue ‘Hyperthermals: rapid and extreme global warming in our geological past’.

## 1. Introduction

In the search for geologic analogues for modern greenhouse gas-driven warming, the Paleocene–Eocene Thermal Maximum (PETM, approx. 56 Ma) has merited attention as the most recent example of rapid carbon release [1–3], because it is the rate of current and projected future warming that makes anthropogenic climate change particularly challenging for natural ecosystems [2,4–6]. The PETM is the most well-known and well-studied example of a ‘hyperthermal event’, characterized by what is often termed a ‘geologically abrupt’ negative carbon isotope excursion (CIE) of greater than 2‰, global warming of 5–7°C and ocean acidification, all of which point to massive carbon release to the atmosphere and/or oceans [7–11]. This rapid and extreme warming at the Paleocene–Eocene boundary occurred against the backdrop of a multi-million year warming trend within a global greenhouse characterized by reduced latitudinal surface temperature gradients and ice-free poles [12–14]. The PETM CIE occurred at the approximate mid-point of a multi-million year declining trend in the δ^13^C of benthic foraminifera following the Paleocene Carbon Isotope Maximum at approximately 57 Ma when δ^13^C reached the highest values of the Cenozoic [14–16]. As the PETM and its temporal context have drawn greater scrutiny, numerous smaller ‘hyperthermals’ have been identified and correlated globally from the Late Paleocene through Middle Eocene, though the PETM CIE stands out as approximately double the magnitude of the next largest event in the sequence and other, smaller events are typically more symmetrical in shape [17–20].

Significant disagreement remains over the source, mass and rate of carbon released during the PETM. Sources are often identified by their δ^13^C signature, which suggests a range of possible masses of carbon release when combined with the magnitude of the CIE. An additional metric is thus required to differentiate sources—temperature change, seafloor carbonate dissolution and surface ocean pH decline have all been used [10,21–23]. However, the timing of carbon injection impacts the assumptions usually made in calculating PETM carbon sources. In order to not conflate uncertainty over timing with uncertainty over the source of carbon, I will herein explicitly discuss the onset duration as opposed to the rate of carbon injection. In one of the earliest attempts to constrain the mass of carbon release responsible for the PETM CIE, Dickens *et al*. [24] noted that an isotopic mass balance approach was valid assuming that carbon release was long relative to the mixing time of the oceans (approx. 10^3^ years) but short relative to the residence time of carbon in the ocean (approx. 1.5 × 10^5^ years). While consistent with the earliest estimates for the onset duration of the PETM CIE (defined as the time interval between pre-PETM and minimum δ^13^C) [12], subsequent estimates for the onset duration have spanned a much broader range, from instantaneous to greater than 20 kyr [25–34]. As a result, disagreement over time scale continues to complicate attempts to constrain the source of PETM carbon. The PETM exemplifies the difficulty of constraining rapid rates of change using the geologic record [35]—an issue that hampers the utility of this and other similar events as analogues for the future. Here, I review the history of estimates for the PETM onset duration before summarizing a series of recently proposed, age-model-independent methods to constrain onset duration. Finally, I use the intermediate complexity Earth system model cGENIE to provide a comparative illustration of PETM scenarios with various assumed onset durations.

## 2. History of Paleocene–Eocene Thermal Maximum onset duration estimates

The PETM CIE was first measured in foraminifera from Ocean Drilling Program (ODP) Site 690 in the Atlantic sector of the Southern Ocean, with an estimated onset duration of 6 kyr in thermocline taxa and 11 kyr in surface-dwellers [12]. These estimates were based on identification of two magnetochron boundaries above and below the CIE and the assumption of constant sedimentation of 1.23 cm kyr^−1^. As the CIE was identified at additional sites in the Atlantic and Pacific, it became clear that isotopic changes corresponded to a dissolution interval, which would have decreased sedimentation rates and invalidated the assumption of constant sedimentation between fairly widely spaced biostratigraphic or magnetostratigraphic tie points [36]. Consequently, Thomas & Shackleton [36] employed an approach that would become common in the development of age models for PETM sites by revising ages to reflect lower sedimentation at sites with severe dissolution using the ODP Site 690 δ^13^C record as a target (given that this site had relatively higher sedimentation rates and less severe carbonate dissolution).

Within the first few years of its discovery, the significance of an extremely rapid onset for the PETM CIE was realized. Given the large magnitude of the CIE (greater than 2‰), if the approximately 10 kyr onset was correct and the cause of the CIE was atmospheric CO_2_ injection (e.g. the Suess effect), then rates of carbon emissions could have been similar to anthropogenic values [37]. At the same time, Dickens [37] emphasized that the 10 kyr onset time scale was tenuous and could be the result of incorrectly applying linear sedimentation rates constrained by tie points across much longer durations than the CIE itself and failing to account correctly for dissolution. Expanded shallow marine and terrestrial records thus seemed a plausible target for correctly constraining the onset time scale. The first identification of the PETM CIE from terrestrial sections was in the Bighorn Basin in North America, with the CIE reported as occurring in 50 kyr. This age model had just four constraining tie points (one of which was the CIE itself) separated by approximately 1–2 Myr intervals [38]. Shortly thereafter, an expanded record of the PETM onset from the Alamedilla section in southern Spain (paleo-Tethys), with chronology based on foraminiferal biostratigraphy and δ^13^C stratigraphy compared to ODP Sites 690 and 865 (equatorial Pacific), was reported to show a slow negative δ^13^C shift over 300 kyr followed by an abrupt CIE with an onset on the order of 10^3^ years [39]. Thus for about the first decade after identification of the PETM CIE, determination of the onset time scale was largely based on low-resolution sedimentation rate estimates derived from microfossil datums, magnetic reversals and δ^13^C chemostratigraphy.

The first cyclostratigraphic age model for the PETM was based on recognition of the precession cycle in measurements of iron (Fe) intensities and magnetic susceptibility within the CIE measured at ODP Site 1051 in the North Atlantic and later refined by combining Sites 1051 and 690 [40,41]. The original cyclostratigraphic age model for Site 1051 identified the occurrence of approximately 1 precession cycle between the base of the excursion and the point at which minimum δ^13^C was reached (the onset duration), with much of the δ^13^C shift occurring in a few thousand years or less. Site 690 cyclostratigraphy suggested that approximately two-thirds of the CIE occurred in two steps that were each less than 1 kyr in duration. The remaining one-third of the excursion then developed over 52 kyr [41].

These and other cyclostratigraphic age models reliant on precession generally make a few key assumptions—that the precession cycle has a 21 kyr average duration (there are periods of 19 and 23 kyr) and that sedimentation is constant within a precession cycle. Thus, cyclostratigraphic methods still involve sedimentation rate calculations, albeit with finer age control in comparison to biomagnetostratigraphy. Additionally, orbital chronologies crucially depend on the correct identification of sedimentary cycles, a difficult proposition across a major dissolution interval [17,37,42]. Others have applied cyclostratigraphy to terrestrial and shallow marine sections—avoiding the truncation common in deep-sea records, but inheriting new challenges, for example due to changes in the carbon source used to reconstruct δ^13^C or uneven sedimentation due to hydrological cycle changes associated with climatic warming [43–45]. An expanded marine succession from the paleo-Tethys (Forada) showed the initial δ^13^C decline over 12.5 cm, equating to approximately 5 kyr based on an approximately 50 cm precession cycle [46]. Cyclostratigraphy on both outcrop [47] and cores [48] from the PETM in the North American Bighorn Basin suggested that the onset occurred within a single precessional cycle (approximately within one-half of a precession cycle) [48]. The Bighorn Basin orbital chronology is broadly consistent with onset duration estimates based on an age model that assumed dynamic sedimentation as a function of the depositional environment [49]. Similarly, independent cyclostratigraphy on a shallow marine section from the paleo-Tethys at Zumaia demonstrated that the majority of the PETM CIE occurred within approximately 5 kyr with the remainder occurring over a single precession cycle [50]. By contrast, tuning using Fe counts across a PETM section from Spitsbergen in the Svalbard Archipelago suggested the onset of the PETM CIE in bulk marine organic carbon occurred gradually over a full precession cycle [51].

A few studies of PETM sections have hinted at even shorter onset duration. First, single foraminifera records from ODP Sites 690 and 865 showed no ‘intermediate’ or transitional δ^13^C values during the onset of the PETM CIE [29,52]. This is not solely a feature of slowly accumulating deep-sea sections, because even single foraminifera records from the North Atlantic continental margin fail to show these so-called intermediates [28,52,53]. While changes in carbonate production and/or preservation are likely to impact these records [25,28], one initial interpretation from these data was that the CIE was effectively instantaneous (less than 500 years) [28,29]. Next, sedimentary couplets across the PETM CIE onset in another record from the North Atlantic continental margin were interpreted as seasonal cycles, with the inference that the onset took 13 years. While interpretation of this record remains highly controversial [32–34,54,55], the lack of intermediate δ^13^C values in particular has led to descriptions for the CIE onset duration as being over several hundred or thousands of years (a shorter estimate than those provided by cyclostratigraphic methods) [56].

**Figure 1.**
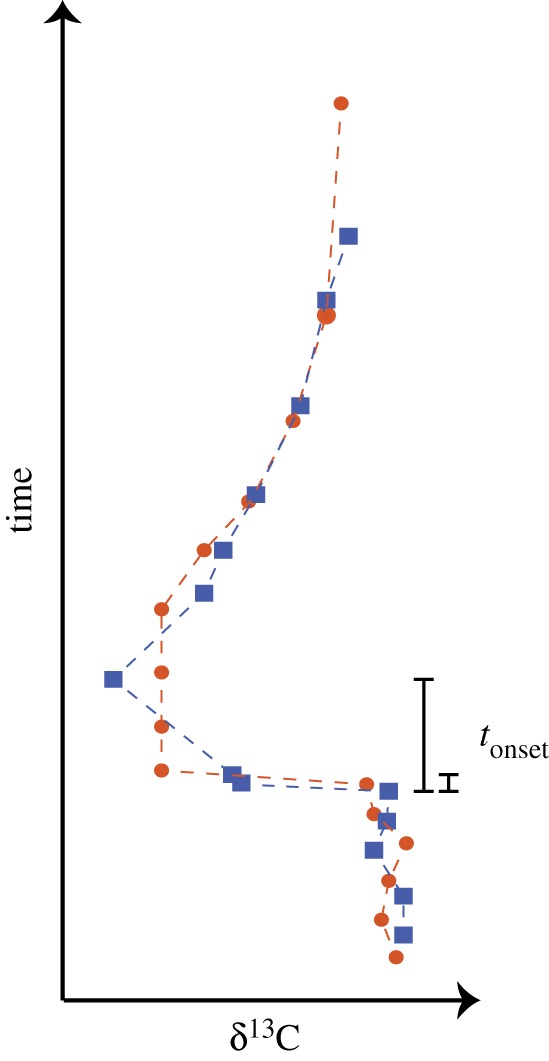
Cartoon demonstrating the definition of the onset duration of the PETM CIE, *t*_onset_, as the time between pre-excursion and minimum δ^13^C. The two possibilities shown indicate the complexity of defining onset time scale if there were multiple, distinct steps in the decline of δ^13^C.

The occurrence of steps in the initial abrupt δ^13^C decline from multiple sites (shallow and deep marine and terrestrial) has led to the suggestion that the initial carbon release may have occurred in pulses and slightly complicating the identification of an onset duration (e.g. figure 1) [42,57–59]. Bowen *et al*. [49] suggested, on the basis of a distinct negative δ^13^C excursion preceding the main CIE in a terrestrial record, that the PETM onset duration was composed of two steps, each developing over approximately 1.5 kyr. There is no single agreed on ‘shape’ for the PETM CIE [11,56], though a few general features have been recognized at multiple sites—these include an abrupt onset (possibly occurring in multiple steps) [49,57,58], a relatively extended ‘body’ of low δ^13^C values lasting for tens of thousands of years [60–62], and a relatively rapid recovery phase [9,50,63] for a total duration of approximately 200 kyr [48]. Based on direct geological time constraints, the argument of Sluijs *et al*. [26] that the PETM onset duration is constrained only between ‘geologically instantaneous' and 50 kyr seems a valid summary.

## 3. Onset duration in Paleocene–Eocene Thermal Maximum modelling experiments

A range of modelling methods have been applied to simulate the PETM by transient carbon input—from carbon cycle box models representing various components of the exogenic carbon cycle to intermediate complexity Earth system models. These experiments are distinct from fully coupled global climate model experiments that attempt to constrain the surface climate response to the PETM. The latter simulate a steady-state climate at elevated atmospheric CO_2_ and therefore make no explicit assumptions about the PETM onset duration (e.g. [64]). Consequently, I focus on experiments that simulate transient CO_2_ input.

As mentioned previously, Dickens *et al*. [24] took the sedimentary constraints as indicative that carbon input occurred slowly relative to ocean mixing time but rapidly relative to the carbon residence time, which justified using a carbon isotope mass balance approach to constrain the mass of carbon injected. Dickens *et al*. [65] employed the Walker & Kasting carbon cycle box model [66] tuned for the present-day carbon cycle and modelled the PETM as an injection of carbon over 10 kyr. With this input time scale, and consistent with the reasoning of Dickens *et al*. [24], the simulated δ^13^C of each reservoir in the model (atmosphere and ocean boxes) behaved similarly, reaching a minimum after 10 kyr. Application of the long-term ocean–atmosphere-sediment carbon cycle reservoir model LOSCAR simulated the PETM onset over 5 kyr (though also including a slow ‘leak’ of carbon over 50 kyr to simulate the extended body of the CIE) while the first applications of the intermediate complexity Earth system model GENIE to the PETM simulated a single uniform pulse of carbon over 10 kyr. Both these models (LOSCAR and GENIE) track the carbon isotopic composition of the atmosphere and oceans in response to carbon input [67,68]. The scenario suggested by Zeebe *et al*. [23] has been modified to reproduce features of the CIE body [69] and recovery [70], though without modifications to the onset time scale. By contrast, addition of a dynamic methane hydrate capacitor to an exogenic carbon cycle box model by Dickens [71] assumed an extended onset duration for the PETM by simulating warming of the deep ocean over 20 kyr and demonstrating that this would drive the release of carbon from a methane hydrate reservoir (causing a CIE).

Another approach has been to model the PETM with instantaneous (single year) carbon release [21,72,73]; for the relatively higher resolution of the University of Victoria Earth System Climate Model (UVic ECSM), this approach is reasonable given the cost of running simulations longer than approximately 10 kyr. However, these simulations will tend to overestimate the increase in atmospheric CO_2_. Meissner *et al*. [21] also tested an onset duration of 4.5 kyr, though this is still relatively short compared with the onset duration assumed by other modelling studies. Additionally, the UVic experiments did not include carbon isotopes and instead focused primarily on comparison to the PETM temperature anomaly and dissolution event.

A number of studies have attempted to directly model individual site records rather than employ simplified scenarios as described in the experiments above. In another application of the GENIE model to the PETM, Cui *et al*. [27] applied a cyclostratigraphic age model developed for the shallow marine PETM record at Spitsbergen to define an approximately 21 kyr onset duration for the CIE [51]. Similarly, the GENIE model (in the cGENIE variant) has been used to calculate carbon input scenarios when forced to follow chronologies developed for ODP Site 401 (constrained by the ODP Site 690 orbital chronology) [9] and the Zumaia section in the Paleo-Tethys [50]. Bowen *et al.* [49] used a carbon cycle box model forced with approximately two 1.5 kyr pulses of carbon input to reproduce key features of a terrestrial carbon isotope record from the Bighorn Basin.

More recently, a series of modelling studies have proposed age-model independent methods for constraining the onset duration of the PETM [25,30,34]. The basic idea is to start with the assumption that no PETM age model developed for an individual site accurately constrains the onset duration of the PETM CIE and ask instead what characteristic model results follow given assumptions about the onset duration. These methods rely (i) on the likelihood of preserving intermediate values during the onset of the CIE from single foraminifera records [25], (ii) on the relative size of the CIE recorded between reservoirs [30], and (iii) on the synchronicity of evidence for warming and the CIE [34]. Respectively, these methods concluded that the PETM onset duration was less than 5 kyr, less than 3 kyr or greater than 4 kyr. In the following section, I describe each of these three methods in greater detail and suggest that these apparently disparate estimates for onset duration are, in fact, reconcilable.

## 4. Age-model independent methods for constraining Paleocene–Eocene Thermal Maximum onset duration

### (a) Method 1: lack of intermediate values in the carbon isotope excursion onset

**Figure 2.**
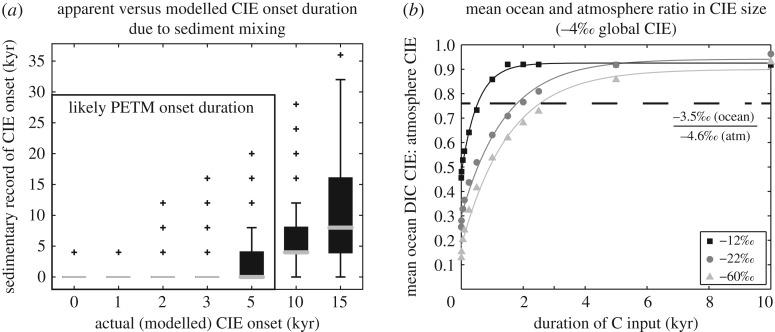
Constraints on PETM onset duration using Methods 1 and 2 described in the text. (*a*) Sediment mixing model constraints on the PETM onset duration. Box and whisker plots indicating the likelihood of the sedimentary record preserving an apparently instantaneous onset (*y*-axis) for a range of modelled onset durations (*x*-axis), with sedimentary onset duration (*y*-axis) defined by the lack of intermediate value δ^13^C individuals modelled in the PETM onset. To determine the modelled onset duration, five individuals were sampled every 2 cm. For each box plot, the lower and upper edges correspond to the 25th and 75th percentile and the median is indicated by the grey line. Whiskers span ±2.7 s.d. and data lying beyond are shown as crosses. The black box indicates that apparently instantaneous onsets are more than 50% likely only for onset duration less than 5 kyr. (Modified from Kirtland Turner *et al*. [25] fig. 6.) (*b*) Ratio of the mean ocean δ^13^C excursion size to the atmospheric δ^13^C excursion size as a function of the duration of carbon input of 14578 Pg C at −12‰ (black squares), 6856 Pg C at −22‰ (dark grey circles) and 2275 Pg C at −60‰ (light grey triangles). Dashed black line indicates the ratio calculated assuming a −3.5‰ mean ocean δ^13^C excursion after McCarren *et al*. [75] and a −4.6‰ atmospheric δ^13^C excursion after Kozdon *et al*. [76] and Diefendorf *et al*. [77]. (Modified from Kirtland Turner & Ridgwell [30] fig. 4*a*).

Arguably the strongest evidence for a geologically instantaneous PETM onset comes from single foraminifera records lacking intermediate or transitional δ^13^C values [28]. Kirtland Turner *et al*. [25] suggested a constraint of less than 5 kyr for the PETM onset duration through application of a sediment mixing model [74] to simulate an individual foraminifera record of the PETM CIE like that generated from ODP Site 690 (figure 2*a*). Directly simulating the dynamics of the sedimentary record through representation of bioturbation, individual ‘particles’, and changes in the relative abundance of various sediment particle ‘types’ allowed the application of a probabilistic method for determining the onset time scale.

This study investigated the likelihood that a CIE occurring over a given duration would appear instantaneous (as in, no intermediate δ^13^C values would be recorded) in the simulated sedimentary record given limited sampling of individual ‘particles’. Parameter values for the sediment mixing model were tuned to represent Site 690 [78] and the model sampling intensity of five individual particles per sample was comparable to that used to generate the single foraminifera δ^13^C record from ODP Site 690 [29]. Kirtland Turner *et al*. [25] concluded that a lack of intermediate values in the Site 690 record was very likely (greater than 50% probability) assuming an onset duration of 3 kyr or less and possible (50% probability) assuming an onset duration of up to 5 kyr (figure 2*a*).

This study represents a way forward in using single foraminifera δ^13^C analyses to constrain the PETM onset duration. Limited sampling is clearly problematic, especially if the abundance of foraminifera declined across the PETM onset. For instance, using the sediment mixing model results for Site 690 but taking larger sampling sizes of 10 individuals instead of five makes it significantly less likely that an event occurring over 5 kyr would appear instantaneous (less than 25% probability compared to 50%). Sampling of 50 individuals would make it extremely unlikely (less than 1% probability) that a 5 kyr onset would appear instantaneous, but would also bias the apparent onset duration towards longer intervals by increasing the likelihood of finding intermediate value individuals mixed up into younger material (Supplementary fig. 14 in [25]). In addition, constraints on changes in the relative abundance of foraminifera taxa, measured on the same size fraction as the single specimen isotope data, are crucial for interpretation, because reductions in abundance will amplify the effects of limited sampling. Finally, sites with high sedimentation rates will show less distortion due to bioturbation (e.g. [78]). Hence, approaches that are capable of measuring the isotopic composition of a large number of single specimens [76], coupled with detailed assemblage data, while targeting sites with relatively high sedimentation rates, present the best opportunity to find and interpret the significance of intermediate value individuals in the PETM onset. These records would also allow further investigation of another interesting aspect of single foraminifera records—the fact that intermediate temperature values are detected on samples with pre-excursion δ^13^C [29]—which may help unravel possible mechanisms of carbon release if preliminary warming was not caused by a carbon source relatively depleted in ^13^C.

### (b) Method 2: relative size of the carbon isotope excursion between reservoirs

On the basis of different CIE magnitudes between reservoirs, Kirtland Turner & Ridgwell [30] suggested the PETM onset could be less than 3 kyr. Kirtland Turner & Ridgwell [30] used the cGENIE model forced with a uniform input of carbon to the atmosphere over durations ranging from 1 year to 10 kyr and compared the relative size of the CIE generated within the ocean and atmosphere as a function of the duration of carbon input (figure 2*b*). Shorter carbon input durations corresponded to larger atmospheric CIEs but not larger mean ocean CIEs. Thus, the ratio of the atmospheric CIE size to the mean ocean CIE size provides a constraint on the duration of carbon input, assuming that the CIE can be accurately measured in each reservoir. For more isotopically depleted carbon sources (and thus smaller masses of carbon released), the modelled atmospheric and surface ocean CIE sizes in Kirtland Turner & Ridgwell [30] were very similar, suggesting a similar relationship between surface ocean and mean ocean CIE size as a function of onset duration. Model results suggested a notable difference in this ratio for carbon input over less than 5 kyr [30].

The difficulty in applying this method comes from accurately interpreting the data to determine the actual size of the CIE in each reservoir. A review of the PETM CIE [8] highlighted the variability of the δ^13^C excursion size recorded globally. Out of 165 compiled carbon isotope records, the mean CIE was −4.7 ± 1.5‰ for terrestrial records and −2.8 ± 1.3‰ for marine records. The smallest CIEs were recorded in marine carbonates and largest in terrestrial plant lipids, mammalian tooth enamel and pedogenic carbonate nodules [8]. There are multiple factors that can bias the CIE recorded in any given reservoir—these include physical or chemical truncation or exclusion of the proxy carrier, post-depositional mixing of either several different contemporaneous carbon sources or material of varying age, or through physiological changes that lead to differences in fractionation by the proxy [11,45,75,77]. However, even when considering potential sources of bias, differences in atmosphere or surface ocean CIE and the mean ocean CIE (as reflected in benthic foraminifera and/or bulk carbonate) remain. The largest CIE measured in benthic foraminifera or bulk carbonate is −3.5‰ [75]. By contrast, measurements of the surface ocean CIE in unaltered planktonic foraminifera revealed a CIE of −4.6‰ [76], consistent with an estimate that attempted to account for changes in plant functional group and climate on fractionation in terrestrial records [77].

A clear way forward in applying this technique is to employ proxy-system models to forward model carbon isotopes in various substrates. Such models would attempt to mechanistically account for transformations from the primary signal in the atmosphere or ocean to the record ultimately measured. Examples are the attempts to model the formation of pedogenic carbonate δ^13^C [49] and carbonate δ^13^C in the marine sedimentary record [78].

### (c) Method 3: synchronicity between carbon isotopes and temperature

On the basis of apparent synchronicity between the high-resolution carbon and oxygen isotope excursions recorded across the PETM onset on the North Atlantic continental margin at Millville [31], Zeebe *et al*. [34] proposed an onset duration for the PETM CIE of greater than 4 kyr. The reasoning was that rapid carbon release (like the decadal time scale proposed for the Milville record [31]) should result in a discernable lag in the rise of sea surface temperature relative to the CIE. Lead-lag determination using the isotope records suggested a delay of no more than two data points (sampled at 0.234 cm spacing). Zeebe *et al*. [34] next compared results from LOSCAR and cGENIE model simulations for the lag between carbon isotopes and temperature as a function of carbon input duration. The models demonstrated that carbon input over 2 kyr would allow a delay of 135 years in the temperature rise, inconsistent with the data, which would allow a lag of just 38 years assuming the entire 24.8 cm onset interval took just 2 kyr. Model and data lags converged for a simulated carbon input over 4 kyr, which further suggested a sedimentation rate of 6.2 cm kyr^−1^ at Millville.

An estimate of greater than 4 kyr suggests no overlap with the onset duration estimate from Kirtland Turner & Ridgwell [30]. However, Zeebe *et al*. [34] compared the North Atlantic shelf records [31] to both modelled global mean sea surface temperature and carbon isotopes in the cGENIE and LOSCAR models as well as to a single grid point on the modelled northwest Atlantic shelf in the cGENIE model corresponding to the paleo-location of the data. Yet the cGENIE model is relatively low resolution (36 × 36 grid) and it is possible that the model site corresponding to Millville does not accurately represent the oceanographic processes relevant for determining leads and lags between carbon forcing and temperature response [32]. Alternatively, though a single model location in cGENIE may provide a questionable basis for comparison, the range in spatial variability reproduced by the cGENIE model may be a more likely indicator of realistic lead-lag time scales as a function of carbon input duration.

To test the range of lead-lag time scales predicted by the cGENIE model in response to a variety of carbon input durations, I used the set of PETM experiments from Kirtland Turner & Ridgwell [30], which simulated carbon input over durations ranging from 1 year to 10 kyr [30]. Here I focus on experiments using a carbon source of −22‰ with a mass of almost 7000 Pg C. This is a greater carbon mass than tested by Zeebe *et al*. [34], but less than the mass calculated by Gutjahr *et al*. [9], and similar to the estimate of Panchuk *et al*. [22], which used the extent of calcium carbonate dissolution to constrain the magnitude of PETM carbon injection. The version of the cGENIE model used was equivalent to that in Zeebe *et al*. [34].

**Figure 3.**
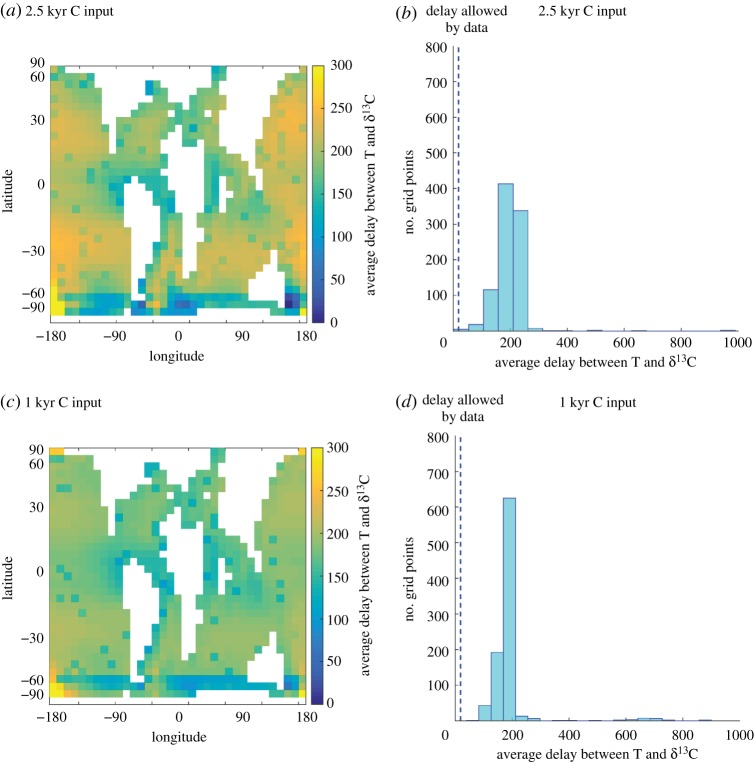
(*a*) Map view of the average delay between the δ^13^C excursion and temperature rise calculated for each site in cGENIE for carbon input over 2.5 kyr calculated using the methodology of Zeebe *et al*. [34]. (*b*) Histogram of the data from (*a*). Dashed blue line indicates the allowable delay from a North Atlantic shelf record of the PETM of 47 years given an onset duration of 2.5 kyr [31]. (*c*) Map view of the average delay between the δ^13^C excursion and temperature rise calculated for each site in cGENIE for carbon input over 1 kyr calculated using the methodology of Zeebe *et al*. [34]. (*d*) Histogram of the data from (*c*). Dashed blue line indicates the allowable delay from a North Atlantic shelf record of the PETM of 19 years given an onset duration of 1 kyr [31].

In order to determine leads and lags from these model experiments, I followed the methodology of Zeebe *et al*. [34], calculating the lead-lag directly from the model normalized responses in surface ocean δ^13^C and temperature. The addition here, compared to Zeebe *et al*. [34], is that I calculated the normalized lead-lag relationship for every ocean grid point in cGENIE, thus characterizing the spatial variability in the lead-lag relationship for a given carbon input duration (figure 3). The intention is to capture the full range of realistic lead-lag time scales given physical processes represented by the cGENIE model.

One question is still whether the simplified physics in the cGENIE ocean model (frictional geostrophic, non-eddy resolving circulation) [79] is sufficient to capture the dynamics of ocean carbon uptake over short time scales. Indeed, this was the criticism suggested by Wright & Schaller [32], who cited the non-uniform response of surface ocean records to the bomb radiocarbon anomaly, which vary as a function of deep-water influence, as a test case for models. On this basis, the cGENIE model is an effective tool for evaluating the spatial response of ocean carbon uptake. Kirtland Turner & Ridgwell [30] simulated the bomb radiocarbon perturbation of the twentieth century in a modern configuration of the cGENIE model (with identical physics to the Paleocene version discussed here) and showed a good model-data agreement with bomb radiocarbon records (their fig. 9).

The results of this spatial lead-lag analysis in cGENIE demonstrate the effect of local oceanographic controls on the disequilibrium between atmosphere and sea surface. Figure 3*a* shows the average delay from the 2.5 kyr carbon pulse experiment in both map view and as a histogram. Most sites have an average delay that falls around 200 years (mean = 193 years). Following the Zeebe *et al*. [34] data-model comparison methodology, if the onset duration at Millville occurred over 2.5 kyr, than the lag between the carbon and oxygen isotopic records of two data points is equivalent to just 47 years. This is clearly much shorter than the typical delay for a 2.5 kyr carbon input calculated in cGENIE. However, there are locations where the lag is shorter (note the overlap between the dashed blue line and binned data in figure 3*a*), notably around continents and in the Southern Ocean. Some sites show delays as short as the 47 years required by the Millville record. The minimum delay recorded at any site is just 22 years.

By contrast, figure 3*b* shows the results of carbon injection over just 1 kyr. In this case, there is no overlap in the lag between carbon and oxygen isotopes allowed by the Millville record (19 years) and the range of lags generated in the cGENIE surface ocean (minimum 64 years). In other words, modelled carbon injection of 1 kyr is inconsistent with the data while carbon injection of 2.5 kyr (using a mass of approximately 7000 Pg C) is allowable by the data. This heterogeneity in the spatial response was hinted at by Zeebe *et al*. [34] in their fig. 4, which shows the delay between the global average δ^13^C and temperature as well as the results for the modelled Milville location. It is also important to note that the global average results presented in [34] are not strictly comparable to the average global delay reported here. In that study, the delay is calculated from global average records of δ^13^C and temperature calculated at each time step, whereas here, the average global delay refers to the mean of all sites' average delay time each calculated individually.

Clearly, more records with resolution comparable to the Millville record would allow firmer constraints on the lead-lag time scale between δ^13^C and temperature across the PETM onset. Yet the application of the Zeebe *et al*. [34] methodology to the full cGENIE model grid allows for a reconciliation of the three age-model independent methods described in detail here, suggesting a lower bound of approximately 2.5 kyr rather than approximately 4 kyr for Method 3, thereby overlapping the range proposed by both Methods 1 and 2.

## 5. Comparison of Paleocene–Eocene Thermal Maximum onset scenarios

Currently, models of the PETM transient carbon input are not directly comparable because no attempt has been made to use consistent conditions for the pre-PETM carbon cycle nor to employ the same carbon input scenario in multiple models as a control. Modelled scenarios vary not only in the assumed onset duration but also in the total mass of carbon released across the event, therefore employing different carbon sources (represented by the δ^13^C of the input). In order to illustrate existing model scenarios for the PETM onset duration, I here adapt a number of proposed PETM scenarios to a simplified, low-resolution version of the cGENIE Earth system model. This configuration uses a Paleocene carbon cycle with temperature-dependent silicate and carbonate weathering feedback enabled as in Kirtland Turner & Ridgwell [30] but has half the spatial resolution (18 × 18 grid) and a simplified continental configuration with a single pole–pole continent. These modifications to the grid allow for substantially increased run-time, but mean that results are not directly comparable between these low-resolution experiments and simulations using the 36 × 36 Paleocene configuration. However, the purpose here is not to provide firm constraints on the magnitude of atmospheric CO_2_ change, temperature rise, pH decline or any other aspect of the event, but rather to illustrate the relative differences between proposed scenarios.

**Figure 4.**
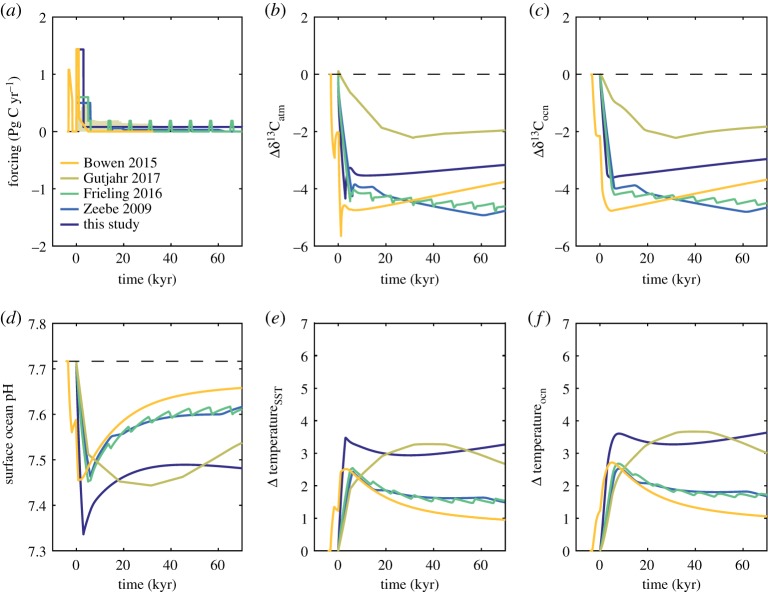
cGENIE model results of the PETM scenarios listed in table 1. (*a*) Carbon forcing (Pg C yr^−1^) (*b*) δ^13^C excursion size in the atmosphere. (*c*) Mean ocean δ^13^C excursion size. (*d*) Surface ocean pH. (*e*) Temperature change in the surface ocean. (*f*) Mean ocean temperature change.

Table 1 includes a description of each PETM carbon input scenario, the carbon input (in Pg C yr^−1^) is plotted in figure 4*a*, and results of these scenarios in cGENIE are shown in figure 4*b–f*. I compare the changes recorded in mean δ^13^C in the atmosphere (*b*) and ocean (*c*), surface ocean pH (*d*) and changes in SST (*e*) and mean ocean temperature (*f*) for each scenario. My focus is particularly on the onset duration of the PETM, so I focus on just the duration over which carbon is emitted in these scenarios (approx. 70 kyr maximum) and exclude the representation of enhanced removal of ^13^C-depleted carbon that could account for the relatively rapid recovery in δ^13^C following the body of the CIE [9,50,63]. I add one further PETM carbon input scenario for comparison to the existing published scenarios (dark blue lines in figure 4): this combines short onset duration (3 kyr) for the input of isotopically depleted carbon (δ^13^C = −35‰) with an extended interval (70 kyr) of elevated rates of volcanic outgassing (δ^13^C = −6‰). The purpose is to illustrate the possibility that the PETM was the result of combining volcanic CO_2_ input of mantle isotopic composition with a pulse of ^13^C-depleted carbon.

**Table 1.** PETM carbon input scenarios.

experiment name	C source used (‰)	total C input (Pg C)	onset duration (kyr)
Bowen 2015	−55	3284	∼3
Gutjahr 2017	avg. −11	10 200	∼21
Frieling 2016	−50 and −45	4500	5
Zeebe 2009	−50	4500	5
this study	−35 and −6	9660	3

These experiments differ not only in the assumed onset duration, but also in the total mass of carbon released, given that a range of carbon sources (identified by their δ^13^C) have been assumed [23,65,69] or calculated [9]. This complicates comparisons, but a few general observations are still possible. First, only short onset durations will lead to an amplification of the CIE size in the atmosphere compared to the mean ocean [30]. This is most obvious with the ‘Bowen 2015’ scenario (yellow lines) as well as the new scenario presented here (dark blue lines) (figure 4). Next, the isotopic composition of carbon released during the extended interval of carbon input required to generate a body to the PETM is not well constrained. δ^13^C values remain low in these experiments because of the slow time scale of weathering and its relative ineffectiveness at restoring δ^13^C. Only scenarios with extended carbon release, however, can prevent a considerably more rapid recovery in surface ocean pH and temperature. Detailed analysis of records of δ^13^C, temperature, and pH from multiple sites is needed to resolve which scenario (if any) best matches all trends. However, it is also important to assess the representation of simulated negative carbon cycle feedbacks (both weathering and C_org_ burial) in order to have confidence about the model results for the recovery time scale of each variable in response to the various proposed carbon input scenarios.

Constraining the onset duration has two major implications: first, it would enable a more apt comparison with modern and future climate change and second, it would provide a strong constraint on the likelihood that surficial carbon cycle feedbacks contributed significantly to carbon release and warming across the event. A rapid onset to the PETM strongly suggests that the initial carbon release was from highly ^13^C-depleted carbon (see Extended Data fig. 5 in Gutjahr *et al*. [9]). This is because a much larger mass of carbon released at the event onset would cause extreme ocean acidification and temperature rise to a degree not yet observed in any PETM records. With constraints on (i) the onset duration, (ii) the mass of carbon released and (iii) the total duration of carbon release, it would be possible to deduce the isotopic composition of the initial carbon input and thus come much closer to constraining the source of carbon for the PETM CIE.

## 6. Conclusion

After more than 25 years of intense study, the PETM continues to be the best analogue for future CO_2_-driven global warming. However, the aspect of the PETM that is most relevant for understanding future impacts—the duration of carbon release—is extremely challenging to constrain using the typical methods for determining age in the geologic record (biomagnetostratigraphy and cyclostratigraphy). Combined data and modelling studies offer a potential way forward by suggesting simulacra of the traces left in the geologic record that indicate a short carbon input duration. Each of the age-model independent methods outlined here has caveats in its application; however, a consensus appears to be emerging that the carbon emissions that drove the CIE occurred over just a few thousand years. This still suggests emissions rates about 10× slower than the current annual average, but is similar to predicted rates of additional carbon release from natural carbon cycle feedbacks [80]. A key remaining challenge for the PETM, however, is in separating the total contribution of carbon from volcanic sources (either at a typical mantle value of −6‰ or more depleted values associated with sill intrusion), from true surface carbon cycle feedbacks.

## Data accessibility

The experiments presented in this paper were all run using the cGENIE Earth system model. Model code is available at https://github.com/derpycode/cgenie.muffin.
